# Path integration in Alzheimer’s disease: orientation, movement and theta rhythmicity

**DOI:** 10.1146/annurev-neuro-102124-020226

**Published:** 2026-04-16

**Authors:** Andrea Castegnaro, Misun Kim, Katarzyna Rudzka, Neil Burgess

**Affiliations:** 1 UCL Institute of Cognitive Neuroscience, University College London, UK; 2 UCL Queen Square Institute of Neurology, University College London, UK

**Keywords:** dementia, angular path integration, head direction, cholinergic modulation, theta rhythm, Alzheimer’s diagnosis

## Abstract

Path integration, the ability to keep track of position and orientation from self-motion, is a sensitive cognitive marker of Alzheimer’s disease. While entorhinal grid cells are central to path integration (PI), we focus here on the broader functional circuit supporting PI and the impact of Alzheimer’s disease within it. This circuit includes orientation from head-direction cells, landmark-based error correction, and signals encoding current or intended movement direction, which we suggest may rely on theta-modulated directional cells and “theta sweeps” in grid and place cell firing. The early vulnerability of PI, particularly angular PI, may reflect multiple sources: pathology in the anterodorsal thalamus degrading head-direction coding; disrupted theta rhythmicity and thus theta-modulated directional signals, potentially reflecting cholinergic dysfunction; and retrosplenial landmark-resetting failures allowing angular drift. We advocate for further cross-species investigation of PI tasks with electrophysiological measures to fully identify the underlying circuit mechanisms and their impairment in Alzheimer’s disease.

## Introduction

Alzheimer’s disease (AD) is the leading cause of dementia (Scheltens et al., 2016) characterised by extracellular β-amyloid plaques and intracellular neurofibrillary tangles of hyperphosphorylated tau, neuronal loss, and cognitive decline (Braak and Braak, 1991; Drummond and Wisniewski, 2017). Recent AD immunotherapies (Sims et al., 2023; Van Dyck et al., 2023) have demonstrated amyloid clearance, but yield modest clinical benefit, potentially because treatment begins after extensive neuronal loss (Høilund-Carlsen et al., 2024). Emerging plasma biomarkers enable scalable, cost-effective detection of pathology before clinical symptoms (Grande et al., 2025), redefining AD diagnosis to include asymptomatic people (Jack Jr. et al., 2024). However, diagnosis based solely on biomarkers is problematic because around 30% of individuals with β-amyloid AD neuropathology did not develop dementia (Stomrud et al., 2015), motivating additional, earlier, and more precise clinical characterisation to optimise intervention (Dubois et al., 2024).

Traditionally, episodic memory decline has been a key cognitive indicator, however it lacks specificity to AD as it also characterises other types of dementia (Sachdev et al., 2014). Path integration, a navigational strategy by which we keep track of our position and orientation by integrating self-motion information, may provide a more sensitive cognitive marker due to the early appearance of tau pathology in entorhinal cortex (Braak and Braak, 1991), which is thought to support path integration (see (Segen et al., 2022)). In contrast to relatively widespread early neocortical amyloid, early tau is focal, spreads trans-synaptically (Braak and Del Tredici, 2011), and correlates more closely with the severity and pattern of cognitive decline (Ossenkoppele et al., 2016).

Grid cells in entorhinal cortex have drawn much attention as a likely neural substrate for path integration (McNaughton et al., 2006; Segen et al., 2022). Here, we focus on the broader circuit supporting path integration, including the functional inputs it requires: (i) head-direction signals for orientation; (ii) velocity signals for movement direction and speed; and (iii) landmark-based error correction for angular and linear estimations. We first synthesise human work – highlighting vulnerabilities in angular estimation – then connect these findings to neural mechanisms via electrophysiological studies in rodents. Finally, we consider how early AD pathology and vestibular alterations perturb these inputs, and outline possible future directions and clinical implications.

### Path integration: linear and angular components

Path integration (PI) is a multisensory computation that continuously updates estimates of position and orientation by integrating two complementary self-motion streams – *linear (translational)* and *angular (rotational)* – derived from vestibular, optic-flow, proprioceptive and motor efference signals (Mittelstaedt and Mittelstaedt, 1982). Although PI can operate in the absence of environmental information, external landmarks play a role in resetting the cumulative errors that inevitably accrue in the integration process.

**Figure 1:**
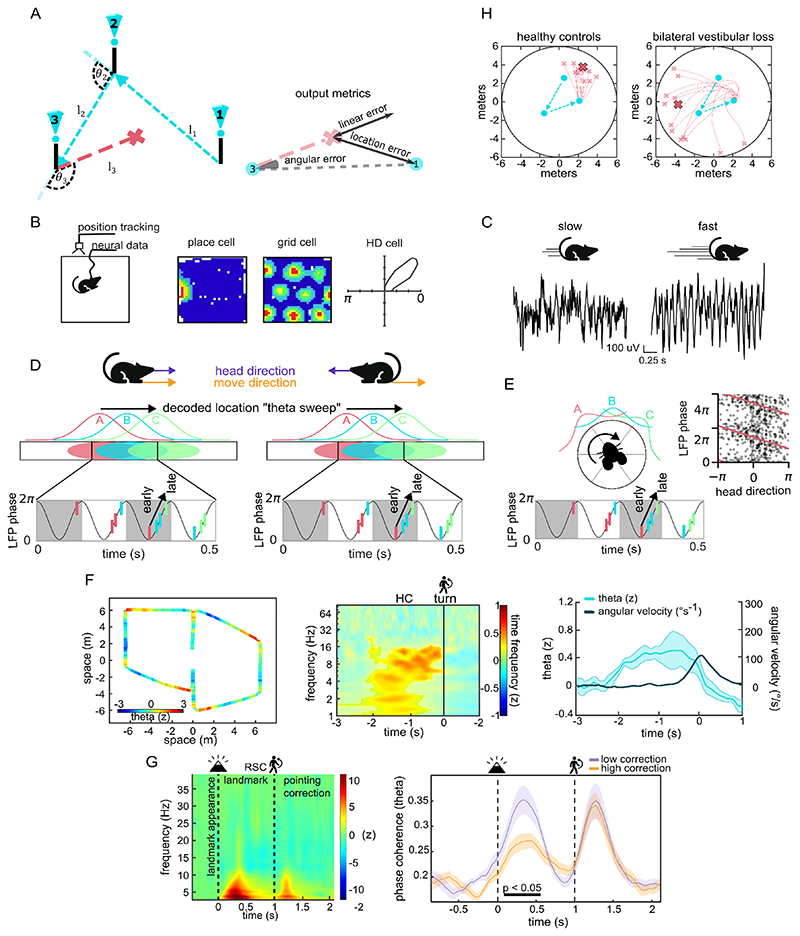
Path integration, neural representations and theta rhythmicity. **A**. Schematic triangle-completion task, adapted from Castegnaro et al., 2023 (CC BY 4.0). **B**. Neural data from foraging rodents. Example firing rate maps from place, grid and head direction (HD) cells, adapted from Barry & Burgess 2014 (CC BY 3.0). **C**. Local field potential (LFP) recorded from the entorhinal cortex in wildtype mice shows the strong effect of speed on the frequency and amplitude of theta oscillation, adapted from Ridler et al., 2020 (CC BY 4.0). **D**. Schematic for theta phase precession and theta sweeps during forward (left) and backward (right) running in linear tracks. Three place cells with firing fields at A, B, and C fire sequentially as the animal moves from A to C. Each cell first fires at a late phase of theta (near the peak), then fires at successively earlier phases (towards the trough) in subsequent theta cycles (coloured ticks overlaid on an 8Hz theta oscillation). At the population level, place cell firing, A (pink)->B (cyan)->C (green) within each theta cycle corresponds to locations sweeping from behind to ahead of the animal, known as “theta sweeps”. The sequential firing pattern reflects movement direction not head direction. **E**. Left: Schematic of theta phase precession in head direction cells during turning, analogous to place cells theta precession during linear movement. Right: An example theta modulated directional cell showing firing (black dots) at progressively earlier theta phase during turning (preferred head direction is aligned to 0°), adapted from Ji et al. 2025b (CC BY 4.0). **F**. Human hippocampal (HC) theta power anticipates upcoming turns, adapted from Seeber et al., 2025 (CC BY 4.0). Left: Z-scored 2–8 Hz theta power in left hippocampus mapped onto the participant’s trajectory. Middle: Time–frequency average across all turns (vertical line = turn onset) revealing a theta bout that precedes rotation. Right: Group-averaged Z-scored theta activity and hip angular velocity showing increased theta activity precedes turns. **G**. Retrosplenial landmark-based error correction, adapted from Naveilhan et al., 2025 (CC BY 4.0). Left: Z-scored time–frequency map in retrosplenial cortex (RSC); one theta peak follows landmark appearance (~300 ms), a second coincides with the physical corrective turn. Right: Theta band (2-8 Hz) inter-trial phase coherence is higher for small angular correction compared to large corrections. **H**. Vestibular loss impairs angular precision in triangle completion. Real-world trajectories from a patient with bilateral vestibular loss versus a healthy control illustrate the patient’s circuitous inbound path and larger angular response variance, adapted from Chari et al., 2023 (public domain).

The triangle completion task is a standard paradigm for assessing PI in humans (Bierbrauer et al., 2020; Howett et al., 2019; Mokrisova et al., 2016) (Fig. 1A). Participants traverse two guided legs (outbound path), before attempting to return to the start (inbound path). Performance is quantified by location error (Euclidean distance between the true start and participant’s endpoint), angular error (difference between the actual participant’s turn and the required turn), and linear error (difference between the inbound distance walked and correct inbound distance). Angular and linear errors are generally uncorrelated; with increasing outbound length, linear errors grow logarithmically, while angular errors remain stable regardless of the geometry or scale of the triangle (Harootonian et al., 2020). When modelling the sources of error, angular and linear errors can show differing dependence on calculation (computing the return trajectory) and execution (enacting the return trajectory) of the inbound path (Chrastil and Warren, 2021). However, caution is needed when distinguishing linear and angular components, since misestimations of either walking distances or turning angles in the outbound path can contribute to both angular and linear errors in the inbound path (Segen et al., 2022). Variants of a triangle task with additional segments increase memory load and cumulative integration errors (Naveilhan et al., 2025), while curved-paths better approximate animal foraging behaviours (Segen et al., 2025).

Aging affects linear and angular PI differently, depending on the type of self-motion information available. In the triangle completion task, which requires the integration of linear and angular components, angular (but not linear) errors explain performance variance in older adults, while both types of error predicted performance in younger adults (Mahmood et al., 2009). When self-motion cues are primarily vestibular (wheelchair), older adults show larger angular errors than controls; when actively walking (vestibular + proprioceptive), age differences diminish (Adamo et al., 2012; Allen et al., 2004). This pattern suggests an age-related vulnerability in angular integration when both components must be integrated and proprioceptive inputs are unavailable.

The angular component has been identified as particularly vulnerable to error accumulation, especially when heading must be inferred from self-motion cues in the absence of external directional anchors (Vickerstaff and Cheung, 2010). Accordingly, heading estimates benefit from reduced variance and reliability-weighted cue integration when landmarks are available, whereas landmark cues have little effect on the variance of distance estimates (Zhang et al., 2020). External landmarks provide reference points to recalibrate one’s internal orientation and prevent the accumulation of error. In featureless settings, heading estimates drift as integration noise accumulates, resulting in progressively larger angular errors (Naveilhan et al., 2025; Vickerstaff and Cheung, 2010). Introducing even a single landmark significantly reduces this drift, as heading direction re-anchors to a stable reference – even when a landmark is only glimpsed briefly during the outbound path (Naveilhan et al., 2025; Zhang et al., 2020).

### Neural basis of path integration: circuit components

#### Spatial cells and path integration

Rodent lesion work indicates a limbic-parietal network for path integration (Frohardt et al., 2006; Maaswinkel et al., 1999; Parron and Save, 2004), consistent with neural correlates of location, direction and speed in these regions; see review (Barry and Burgess, 2014). Hippocampal place cells fire when the animal is at specific locations; entorhinal cortex (EC) grid cells fire at multiple locations tiling the space in a hexagonal pattern; head direction (HD) cells in anterior thalamus, presubiculum, EC and retrosplenial cortex (RSC) fire when animal is facing a specific direction (Fig. 1B). EC speed cells increase their firing rate with running speed. During cue-poor foraging, spatial firing of these cells persists but becomes noisier, and the stability of these correlates with PI accuracy (Gil et al., 2018; Valerio and Taube, 2012). These neural representations of location and orientation complement more perceptual and motoric representations in parietal areas (Alexander and Nitz, 2015; Whitlock et al., 2012). The two systems potentially interface via “gain-field” neurons in posterior parietal or retrosplenial areas characterised by conjunctions of both types of responses (Bicanski and Burgess, 2018).

In humans, intracranial studies report hippocampal place-responsive cells (Ekstrom et al., 2003; Miller et al., 2013) and grid-cell-like neurons in EC (Jacobs et al., 2013; Miller et al., 2015); fMRI identifies place-sensitive signals in the hippocampus (Hassabis et al., 2009; Kim et al., 2017), direction-sensitive signals in the thalamus and RSC (Lu et al., 2025; Shine et al., 2016) and grid cell-like signals in EC (Doeller et al., 2010). Interestingly, rotation and translation dissociate under paradigms that isolate these components: hippocampal/parahippocampal activity predict linear distance memory and RSC activity predicts angular distance memory (Chrastil et al., 2016). This is consistent with the topographic disorientation (“losing sense of direction”) following RSC lesion and increased RSC activity upon recovery (Ino et al., 2007; Kawakami et al., 2024). Importantly, RSC and thalamus activity is tuned to both stable, globally orienting landmarks (Shine et al., 2016) and perceptual self-motion cues (Chen et al., 2024). This flexibility supports the view that RSC integrates PI signals from limbic areas and those derived from visual/perceptual areas (Bicanski and Burgess, 2018).

#### Theta oscillations

The theta rhythm (4-10Hz) is a prominent oscillation in the local field potential of the hippocampus and EC that, in rodents, is tightly coupled to self-motion (Fig. 1C). Locomotion or even intention to move strongly drives theta (Bland and Oddie, 2001). Disrupting the medial septum abolishes speed-modulated theta and degrades grid periodicity (Brandon et al., 2011; Hinman et al., 2016; Koenig et al., 2011). Beyond encoding speed, theta temporally organises spatial information: place and grid cell firing exhibits theta phase coding of distance as the animal traverses their firing fields; see review (Burgess and O’Keefe, 2011) (Fig. 1D). As a result of this mechanism, at the population level, place and grid cells show “theta sweeps” in which the represented location sweeps forwards within each theta cycle (Fig. 1D), potentially sampling future locations (Johnson and Redish, 2007; Kay et al., 2020; Vollan et al., 2025). Theta phase coding has now been observed across mammalian species including humans (Qasim et al., 2021) and flying bats (Forli et al., 2025).

Human recordings show movement-related theta that, although less continuous, appears adapted to both self-motion and task context. Intracranial electroencephalography (EEG) during real-world untethered walking reveals brief bouts of ~8 Hz theta in hippocampal/parahippocampal cortex (Aghajan et al., 2017). In desktop VR, hippocampal theta power increases at movement onset in low (2-5 Hz) and high (6-9 Hz) bands and scales with distance travelled (Bush et al., 2017), with the high band tracking instantaneous speed (Goyal et al., 2020). The balance between theta bands shifts from higher during real walking to lower when vestibular/proprioceptive input is removed (Bohbot et al., 2017).

While physically walking a remembered route, hippocampal theta power peaks ~200 ms before body turns at junctions, and their probability (not amplitude) predicts the upcoming turn (Seeber et al., 2025) (Fig. 1F). However, theta also persist without optic flow: during “teleportation” in VR, low-frequency (3-4 Hz) theta still differentiates travelled distance, implying an internally driven spatial updating mechanism (Vass et al., 2016). Beyond the hippocampal formation, theta synchrony in humans extend across the navigation network: in RSC cortex, theta power scales with vestibular-driven rotational acceleration (Gramann et al., 2021); in a path integration task, landmarks briefly presented during the outbound path to recalibrate angular errors increase RSC theta power, and theta phase-reset indexes the magnitude of angular correction (Naveilhan et al., 2025) (Fig. 1G). Finally, theta coordinates human grid-like representations in EC and ventromedial prefrontal cortex (Chen et al., 2021), offering a circuit mechanism for integrating self-motion with schematic context.

#### Vestibular, directional and theta pathways

**Figure 2:**
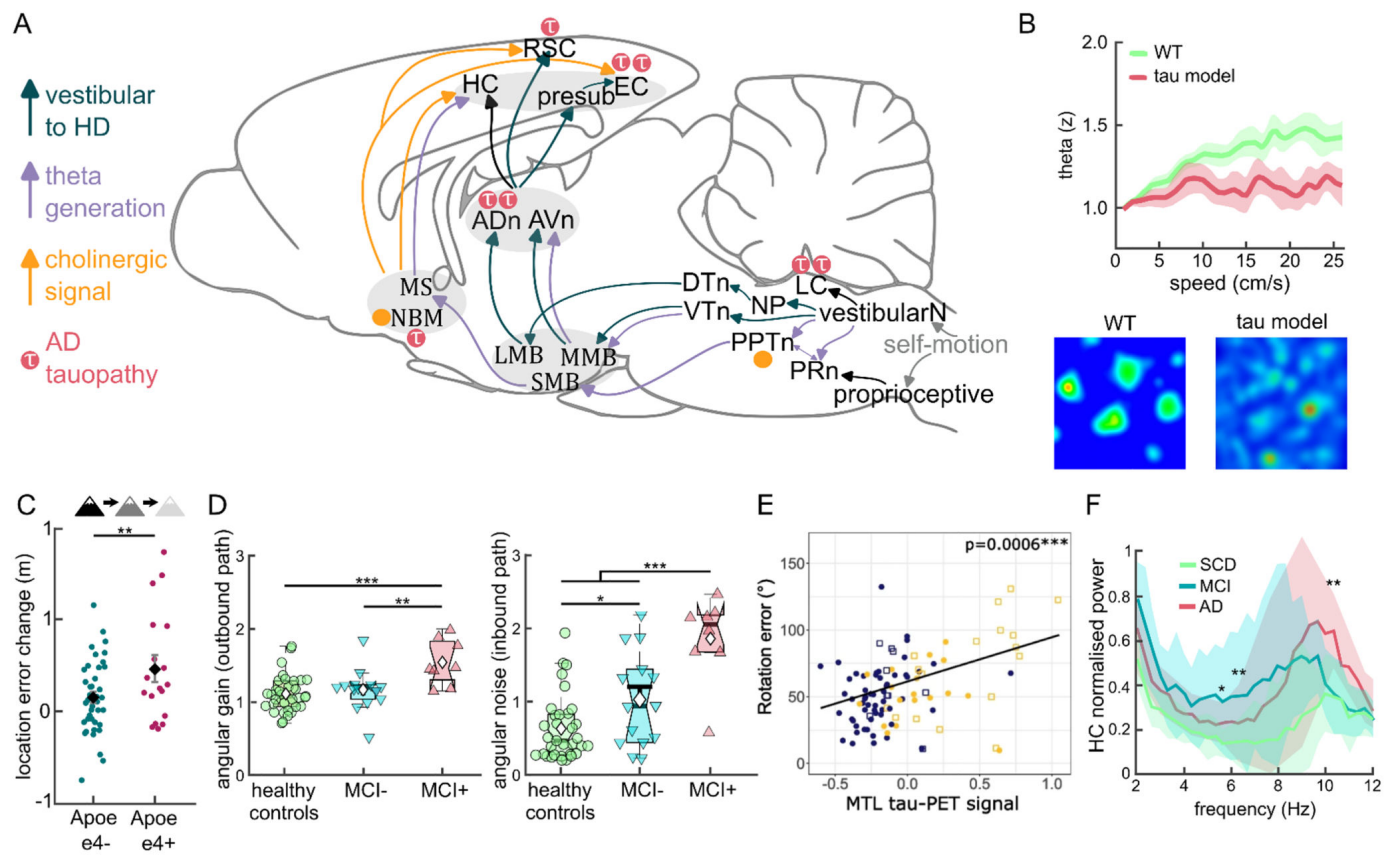
Relevant neural circuitry and functional AD related changes. **A**. Vestibular inputs to HD cell networks (dark green lines), vestibular inputs to theta rhythmic regions (violet lines), and cholinergic inputs to the hippocampal formation (orange lines). Regions showing early tauopathy in AD (pink τ symbol). Abbreviations: VestibularN, vestibular nuclei; LC, locus coeruleus; NP, nucleus prepositus; DTn; dorsal tegmental nucleus; VTn; ventral tegmental nucleus; PPTn, pedunculopontine nucleus; PRn, pontine reticular nucleus; MMB, medial mammillary body; LMB, lateral mammillary body; SMB, supramammillary body; ADn, anterodorsal nucleus of thalamus; AVn, anteroventral nucleus of thalamus; HC, hippocampus; presub, presubiculum; EC, entorhinal cortex; RSC, retrosplenial cortex; MS, medial septum; NBM, nucleus basalis of Meynert. **B**. Top: Theta power does not increase with running speed in tauopathy mice. Bottom: grid cell firing patterns from a wildtype (left) and a tauopathy (right) mouse, adapted from Ridler et al. 2020. **C**. Genetic risk amplifies cue-poor path-integration deficits among people with family history of AD: APOE-ε4 carriers show larger changes from baseline in location error when distal cues are reduced, adapted from Newton et al., 2024 (CC BY 4.0). **D**. Prodromal AD affects angular path integration, adapted from Castegnaro et al., 2023. (CC BY 4.0) Left: individuals with MCI and positive AD biomarkers show greater overestimation of the outbound turn relative to the biomarker-negative MCI and age-matched controls. Right: The same group also shows larger angular noise in the inbound path. **E**. Rotation errors correlate with medial temporal tau burden, adapted from Colmant et al., 2025 (CC BY-NC-ND 4.0). **F**. Source-localised MEG signal shows increased hippocampal theta power in biomarker-positive MCI compared to SCD participants, adapted from Luppi et al., 2022 (CC BY-NC 4.0). Legend: AD = Alzheimer’s disease, MCI = mild cognitive impairment, SCD = subjective cognitive decline; *p < 0.05, **p < 0.01, ***p < 0.001.

When animals turn or move their heads, inertial forces deflect hair cells in distinct vestibular organs – the semicircular canals that detect angular acceleration and the otolith organs that detect linear acceleration – whose signals are propagated to the vestibular nuclei in the brain stem (Khan and Chang, 2013). From there, ascending brainstem-thalamic pathways deliver two essential inputs to the navigation system: i) head-direction signals to presubiculum/entorhinal cortex and ii) a theta drive that paces hippocampal-entorhinal activity (Fig. 2A). First, the vestibular → nucleus prepositus → dorsal tegmental → lateral mammillary → anterodorsal thalamic nucleus (ADn) pathway conveys HD signals to presubiculum and entorhinal cortex; see review (Taube, 2007) while visual input is integrated with HD in the RSC, which has reciprocal connections to the visual cortex, thalamus, and subiculum. Second, ascending vestibular projections target the pedunculopontine tegmental nucleus, a locomotor hub, and the pontine reticular nucleus, which receives neck- and limb-proprioceptive inputs; see review (Aitken et al., 2018). These brainstem relays drive supramammillary and posterior hypothalamic inputs to medial septum (Takano and Hanada, 2009; Vertes and Kocsis, 1997), whose rhythmically firing cholinergic and GABAergic neurons pace theta rhythmicity.

A parallel theta generating route runs via the ventral tegmental nucleus of Gudden (VTN) (Bassant and Poindessous-Jazat, 2001) reciprocally connected with the medial mammillary nucleus, which in turns project to the anteroventral thalamic nucleus (AVn) (Vann, 2009). With broad brainstem connections, including the vestibular nuclei, the VTN acts as a midbrain hub transferring motor and sensory information to limbic circuits (Irle et al., 1984). The anteroventral thalamic nucleus contains theta-modulated direction cells, delivering a theta drive to the directional network that might play a special role in theta sweeps and PI (see next section).

Vestibular processing is especially important for the angular component of PI, and damage to it specifically impairs angular rather than distance replication when walking blindfold (Glasauer et al., 2002). In the triangle completion task, patients with vestibular hypofunction exhibit larger location errors driven by increased angular variance (Chari et al., 2023; Xie et al., 2017) (Fig. 1H). During novel routes, bilateral vestibulopathy reduces the active use of visual cues for reorientation (fewer object fixations, slower head turns) (Schöberl et al., 2021), and even mild, unilateral or partial bilateral loss selectively impairs rotational memory while sparing non-spatial cognition (Anson et al., 2021; Dordevic et al., 2021).

Functional neuroimaging reveals a distributed vestibular–spatial network: reduced hippocampal and entorhinal activation accompanies behavioural errors, whereas parahippocampal and retrosplenial regions show compensatory over-activity in vestibulopathy (Kremmyda et al., 2016; Schöberl et al., 2021). Vestibular dysfunction is often associated with hippocampal volume loss (Brandt et al., 2005; Lee et al., 2023). However, hippocampal atrophy is neither necessary nor sufficient to explain vestibular-related deficits (Dordevic et al., 2021).

#### Theta-modulated head directional cells: an internal movement direction signal?

Grid cells are thought to support path integration as part of a continuous attractor network also involving conjunctive direction x grid cells (Sargolini, 2006), in which velocity input drives the “bump” of neural activity to follow the movement of the animal (McNaughton et al., 2006). A key challenge is that directionally tuned grid cells encode animal’s HD (Sargolini, 2006), not movement direction. Because HD and movement direction can dissociate (e.g. head scanning during locomotion), simulations predict impaired PI when HD, rather than movement direction, is integrated (Raudies et al., 2015). Notably, in Drosophila, the ‘missing’ movement direction component has been found separately to HD cells (Lu et al., 2021), however movement-direction cells remain elusive in mammals.

A recently described “internal direction” signal may bridge this gap. In rats, “theta sweeps” decoded from place and grid cells progress forwards, alternating left-right across theta cycles (Kay et al., 2020; Vollan et al., 2025). This alternation is dissociable from HD and is inherited from the theta-modulated directional cells in parasubiculum. Classic HD cells in the ADn lack theta rhythmic firing (Blair et al., 1999), whereas the directional cells in the AVn are theta-modulated (Ji et al., 2025b; Lomi et al., 2023; Tsanov et al., 2011), consistent with their respective inputs from lateral and medial mammilary bodies (Fig. 1E). Theta-modulated directional signals also appear in the hippocampal formation, including directional (Brandon et al., 2013; Vollan et al., 2025) and conjunctive cells: place×direction (Cacucci et al., 2004); direction×speed and direction×angular speed (Spalla et al., 2022). A network model incorporating firing rate adaptation and theta modulation successfully captures theta phase precession in theta-modulated directional cells (Fig. 1E) and how these cells drive theta sweeps in grid and place cells to follow the internal direction rather than head direction (Ji et al., 2025a, 2025b; Vollan et al., 2025).

This raises the question of whether the internal direction encoded by theta sweeps (and presumably theta-modulated direction cells) dissociates from HD when the two are misaligned. In backward locomotion training on a linear track, HD cells followed HD, whereas hippocampal place cells show theta phase precession, and therefore theta sweeps, relative to the movement direction (Cei et al., 2014; Maurer et al., 2014) (Fig. 1D). In settings where the animal’s desired goal direction is dissociated from movement and head direction, it can be seen that theta sweeps indicate goal direction (Yu et al., 2025). We assume that the home location in triangle completion would similarly be indicated by theta sweeps. Thus, the direction of actual or desired movements could be encoded implicitly by theta phase-based mechanisms, such as theta sweeps, see also (Burgess and O’Keefe, 2011). Alternatively, they might arise outside of the limbic system, such as in parietal cortex which contains cells tuned to self-motion (e.g., forward, left, right) (Alexander and Nitz, 2015; Whitlock et al., 2012).

#### Cholinergic modulation of theta and spatial cells

In rodents, voluntary locomotion elicits hippocampal type I theta (7 – 12 Hz), classically resistant to cholinergic antagonist atropine and strongly modulated by movement speed (Kramis et al., 1975). In contrast, type II theta (4 – 7 Hz) appears during immobility or anaesthesia and is atropine-sensitive. However, this dichotomy is incomplete: cholinergic mechanisms also contribute during movement; see review (Gu and Yakel, 2022), with systemic administration of cholinergic antagonists reducing the speed modulation of theta frequency (Newman et al., 2013), degrading grid periodicity (Newman et al., 2014), and disrupting theta-modulated spiking of place cells while sparing place fields (Newman et al., 2017). Acetylcholine also plays a prominent role in scheduling encoding versus retrieval within the hippocampal formation, linked with different phases of the theta cycle (Douchamps et al., 2013; Hasselmo, 2006). Similarly in humans, pharmacologic cholinergic blockade disrupts low theta (2–4 Hz) amplitude and phase alignment during episodic memory encoding (Gedankien et al., 2023).

Cholinergic influences also diverge for angular versus linear motion in rodents. During passive rotation, angular speed modulates low-frequency hippocampal theta which is dependent on both vestibular and medial septal cholinergic inputs (Shin, 2010; Tai et al., 2012). By contrast, passive linear translation induces hippocampal theta (with lower frequency and amplitude than active walking) whose frequency scales with linear speed, but which remains (at increased frequency) after cholinergic blockade (Xie et al., 2012). The increase in theta power and frequency from passive to active translation is consistent with the effect of shifting from virtual to real navigation observed in humans (Bohbot et al., 2017). The observation of a cholinergic and vestibular-dependent theta during passive rotation should be tested in active navigation in rodents and humans.

### Early AD pathophysiology and impaired path integration

#### Early pathology in AD

Histological studies indicate locus coeruleus (LC) - the brain’s major noradrenergic hub (Nikolenko et al., 2024) - as a first site of tau accumulation in AD, showing pretangle tau in LC before neurofibrillary tangles appear in entorhinal/perirhinal cortices (Braak and Del Tredici, 2011; Weinshenker, 2018). Along with entorhinal cortex, LC expresses high levels of ApoER2, a receptor in the Reelin signalling cascade involved in regulating tau phosphorylation which can make these regions particularly vulnerable to AD (Ramsden et al., 2023). Notably, the ApoE4 isoform, a genetic risk factor for AD, reduces the surface expression of ApoER2 (Chen et al., 2010), whereas a gain-of-function mutation in this Reelin pathway has been implicated in sparing the EC from tau pathology and supporting cognitive resilience despite otherwise widespread neuropathology (Lopera et al., 2023). Reelin-expressing cells are abundant in layer II of entorhinal cortex where grid cells are found (Igarashi, 2023). Accordingly, diffusion-MRI shows microstructural changes in the LC-entorhinal white-matter track in individuals with prodromal tau pathology and amyloid-negative neurodegeneration (Aiello et al., 2025). Rostral LC projections target medial temporal areas, whereas caudal LC projections target areas involved in sensorimotor and general cognitive processing (Veréb et al., 2023). This subdivision may be differentially vulnerable in AD, however we lack studies linking segment specific LC degeneration to early tau pathology and resulting cognitive outcomes in AD.

Converging evidence also implicates the extended Papez circuit from the earliest stage of AD: diffusion-MRI revealed unexpected degeneration of the anterior thalamic tract on a par with the LC–entorhinal pathway (Aiello et al., 2025), with ADn, part of this pathway, affected by tau at the earliest stage (Sárkány et al., 2024). Given ADn’s role in head direction (HD) coding, angular PI may degrade before broader limbic decline. In mice, HD cells are abundant in ADn, and virally induced tau in ADn causes disorientation and accumulates in ADn terminals within retrosplenial granular cortex, implicating early disruption of thalamic to retrosplenial communication (Jiang et al., 2024). Complementing this, anterior thalamic lesions disrupt both grid and HD cell firing (Winter et al., 2015), predicting degraded entorhinal spatial signals and spatial disorientation, especially angular PI, in early AD. By contrast, directional tuning of HD and conjunctive HD x grid cells recorded in the entorhinal cortex remains relatively intact in transgenic mice in which mutant amyloid or tau develops primarily in the hippocampal formation (Fu et al., 2017; Ridler et al., 2020; Ying et al., 2022). Finally reduced connectivity associated with early tau spread across hippocampus, entorhinal cortex, and posterior cingulate (including retrosplenial) regions in preclinical older adults, underscores the early network disruption in navigation-relevant circuits (Berron et al., 2021; Coughlan et al., 2020; Fischer et al., 2024). Of note, the retrosplenial cortex shows consistent hypometabolism in prodromal AD, and its functional connectivity with the parahippocampal cortex declines early in preclinical stages (Hari et al., 2023; Nestor et al., 2003).

#### How is path integration impaired in AD?

PI deficits are evident in prodromal AD (Howett et al., 2019; Mokrisova et al., 2016), and in at-risk groups such as APOE-ε4 carriers (Kunz et al., 2015), together with reduced grid-like signals (Bierbrauer et al., 2020), volumes (Howett et al., 2019) and functional connectivity in the entorhinal region (Coughlan et al., 2020). In clinically healthy older adults, poorer PI also correlates with phosphorylated tau and β-amyloid levels, suggesting that PI might indicate preclinical pathology (Coughlan et al., 2023a).

In a middle-aged cohort with family history of AD, APOE-ε4 carriers exhibited larger PI errors than non-carriers when distal landmarks are reduced (Newton et al., 2024) (Fig. 2C), consistent with a compensatory shift toward boundary-anchored navigation (Coughlan et al., 2020; Kunz et al., 2015). These results are consistent with attenuated grid-like signals under cue-poor conditions found in similar genetic at-risk group (Bierbrauer et al., 2020). Dissociating angular and linear processing of the outbound and inbound paths in triangle completion isolated two biases characteristic of prodromal AD: outbound angular gain and inbound angular noise, both higher in the biomarker-positive mild cognitive impairment patients (Castegnaro et al., 2023)(Fig. 2D). Complementing this, individuals with preclinical AD showed PI impairments despite intact performance on standard memory tests; critically, angular, but not linear, errors were associated with media temporal tau on PET scanning (Colmant et al., 2025)(Fig. 2E). Finally, large-scale wayfinding analyses successfully distinguish APOE-ε4 carriers from controls using a “curvature” index (characterising circuitous paths and detours) (Lim et al., 2023).

Mouse models of AD show dysfunction in grid (Fu et al., 2017; Jun et al., 2020; Ying et al., 2022) and HD cell firing (Jiang et al., 2024) and reduced medial septal cholinergic neurons (Hampel et al., 2018; Mehla et al., 2019), with associated spatial memory deficits. (We note that the β-amyloid induced models also tend to show tau hyperphosphorelation (Kourti and Metaxas, 2024).) For example, the human APP-NL-G-F knock in mouse (Saito et al., 2014) shows age-related cognitive deficits from 6-8 months (Masuda et al., 2016; Mehla et al., 2019) with impaired grid cell firing (Jun et al., 2020). Impaired grid cell firing could originate from disrupted speed coding in theta oscillation in tauopathy (Ridler et al., 2020) (Fig. 2B) and in amyloidopathy (Cayzac et al., 2015). AD mice also exhibit reduced theta phase modulation and spatial information coding (Cheng and Ji, 2013; Mably et al., 2017; Wood et al., 2023). However, the observed effects vary across models, and the changes responsible for specific impairments in AD require further clarification, for example, theta frequency or power can be reduced (Cayzac et al., 2015; Ridler et al., 2020), unchanged (Viney et al., 2022), or increased (Fu et al., 2017; Jun et al., 2020) in different models.

#### Theta rhythmicity and cholinergic dysfunction

The forebrain cholinergic system is markedly disrupted in AD. Progressive tau pathology in the basal forebrain – especially in the nucleus basalis of Meynert (NBM) (Mesulam, 2013; Van Beek and Claassen, 2011) – drives limbic and neocortical cholinergic denervation (Schmitz and Nathan Spreng, 2016). Receptor profiles also shift: muscarinic M1 receptors are relatively preserved, but cortical nicotinic (notably α7) and presynaptic M2 receptors are reduced (Mash et al., 1985). Functionally, this combination undermines hippocampal plasticity (Tropea et al., 2021), and weakens cholinergic–vascular coupling, impairing cerebral haemodynamic processes (Van Beek and Claassen, 2011).

These disruptions are highly likely to impair path integration. The NBM innervates widespread cortical and medial temporal targets, including hippocampus and entorhinal cortex (Schmitz and Nathan Spreng, 2016). Cholinergic modulation supports theta dynamics and synaptic plasticity that stabilise self-motion representations, therefore altered cholinergic signals in AD can impair speed modulation of theta rhythms and degrade grid cell firing (Newman et al., 2014, 2013). The apparent cholinergic role in rotational theta in rodents (Shin, 2010; Tai et al., 2012), and the association between theta and turning in humans (Gramann et al., 2021; Seeber et al., 2025) might make angular estimation particularly vulnerable.

Increased global theta power is a robust signature in AD (and to a lesser extent in mild cognitive impairment) relative to healthy aging (Musaeus et al., 2018; Rossini et al., 2006), with theta discriminating AD from controls more clearly than other frequency bands (Engels et al., 2016). Magnetoencephalography (MEG) studies similarly report elevated hippocampal theta in prodromal AD compared to subjective cognitive decline, highlighting regional vulnerability of theta rhythmicity early in the disease (Luppi et al., 2022)(Fig. 2F).

#### Vestibular deficits in AD

Finally, prodromal and AD patients show postural instability and reduced vestibular reflexes, indicating subtle vestibular pathway impairment (Birdane et al., 2012; Harun et al., 2016; Leandri et al., 2009). In blindfolded older adults, angular path-integration on a rotational chair enabled above-chance classification of AD genetic risk carriers from accelerometer/gyroscope signals, consistent with vestibular dysregulation (Coughlan et al., 2023b). Vestibular loss may therefore contribute to AD and could serve as an early behavioural marker (Previc, 2013), but its pathophysiology in AD remains to be clarified.

## Discussion

We have reviewed the mechanisms underlying path integration and their links to pathology in AD, with an emphasis on angular estimation which seems particularly vulnerable early in the progression to Alzheimer’s dementia. While grid-cell firing in the entorhinal cortex is tightly linked to PI, our review highlights three components from the broader circuit that are also likely to be important: i) head direction (HD) cells which are necessary for orientation, and the anterodorsal thalamic nuclei (ADn) which are central to the HD circuit; ii) hypothesised signals that encode the direction of current or desired movement, potentially organised by theta dynamics via ‘theta sweeps’; iii) landmark-based error correction, important for correcting homing in path integration, potentially mediated by retrosplenial cortex. Focusing on these inputs, we reframe PI as an interaction of multiple systems, and describe how each system might cause a PI deficit in early AD.

First, orientation: converging histopathology and imaging implicate the extended Papez circuit from the earliest stages of AD. The anterior thalamic tract, and ADn in particular, shows tau vulnerability; tau induction in mouse ADn reduces HD tuning and anterior thalamic lesions disrupt both HD and grid signals, consistent with angular PI vulnerability before broader limbic decline.

Second, current and desired movement direction: beyond classic HD cells, theta modulated directional cells provide a candidate for the movement-direction signal that is required for PI. These cells exhibit theta phase organisation across their directional fields, and drive theta sweeps. Recent research shows that theta sweeps provide an internal direction signal reflecting potential exploratory movements during foraging and goal-directed movements during navigation. Cholinergic dysfunction in AD is a plausible point of PI failure due to cholinergic control of theta: in humans, cholinergic manipulation disrupts low-frequency theta oscillations and phase alignment, while muscarinic blockade in rodents reduces the speed modulation of theta and degrades grid cell firing. Given strong cholinergic control of theta during rotation, angular PI could be more vulnerable.

Third, landmark-based error correction: human and comparative evidence places retrosplenial cortex as a hub combining limbic computations with perceptual inputs from visual and parietal areas. Retrosplenial activity is tuned to stable landmarks and self-motion cues, with its theta power increasing during landmark-based correction in PI. Lesions to retrosplenial cortex are associated with directional disorientation, and early network changes spanning EC-posterior cingulate/retrosplenial cortex in preclinical populations reinforce the vulnerability of this region in AD. Consistent with this anchoring role, simultaneous recordings show near-synchronous head-direction representations in ADn and RSC during cue rotation and in darkness, compatible with a strong feed-forward drive from ADn and thalamo-cortical update of orientation (van der Goes et al., 2024).

Vestibular contributions likely amplify angular vulnerability. In patients and older adults with vestibular hypofunction, larger location errors in path integration are driven by increased angular variance and circuitous homing; neuroimaging reveals compensatory over-activity in parahippocampal/retrosplenial cortex with reduced EC–hippocampal activity in vestibulopathy patients. This is consistent with the vestibular system being the upstream input to theta and cholinergic systems and therefore should be taking into consideration when addressing AD.

Future research requires longitudinal tests of a circuit-level hypothesis: early ADn tau in relation to impaired HD/grid cells and disorientation; cholinergic-theta disruption in relation to degraded movement-direction coding and angular encoding; landmark-resetting failures in AD and how this creates cumulative angular drift. In testing these hypotheses, protective mechanisms could yield additional insights, e.g. Reelin-linked sparing of the entorhinal cortex from tau pathology associated with cognitive resilience (Lopera et al., 2023).

Cross-species paradigms should align task structure (e.g., triangle completion in (Duncan et al., 2025)) to maximise translational validity by facilitating direct comparisons between human and rodent navigation strategies, both for resolving outstanding mechanisms and mapping human AD deficits to AD mouse models. Virtual reality allowing head-rotation (Chen et al., 2018) is well-suited for cross-species studies as it enables experimental control over features – e.g. landmark availability – while preserving angular vestibular input. In humans, portable neurophysiological methods such as EEG (Gramann et al., 2021) and optically-pumped MEG (Boto et al., 2018; O’Neill et al., 2025) combined with VR (Castegnaro et al., 2023; Howett et al., 2019) could track movement-linked theta in naturalistic PI paradigms.

In summary, angular PI deficits in AD may best be understood as failures of inputs to the path integration system – orientation (ADn/HD), movement direction and speed (theta-organised signals which are cholinergic-dependent), and landmark resetting. Targeting these inputs may provide behavioural and neural biomarkers for preclinical detection and for evaluating circuit-informed interventions.
